# The effect of different liquids on paper strength demonstrates the central role of hydrogen bonding

**DOI:** 10.1038/s41598-026-69766-y

**Published:** 2026-09-09

**Authors:** Niklas Kvarnlöf, Gunnar Henriksson, Björn Sjöstrand

**Affiliations:** 1 Department of Engineering and Chemical Sciences, Karlstad University, Karlstad, Sweden; 2 Department of Fiber and Polymer Technology, Wallenberg Wood Science Centre, Royal Institute of Technology, KTH, 100 44 Stockholm, Sweden

**Keywords:** Hornification, Hydrogen bonding, Wet strength, Paper strength, Hofmeister series, Hydrophobic interaction, Chemistry, Materials science, Physics

## Abstract

Paper derives its mechanical integrity from a complex network of interactions between cellulose fibres, yet the relative importance of hydrogen bonding, hydrophobic interactions and electrostatic forces remains debated. Here, the wet tensile strength of paper sheets are investigated when exposed to water, a series of organic solvents with differing hydrogen-bonding characteristics, and aqueous salt and urea solutions designed to modify electrostatic and hydrophobic interactions. By systematically varying the liquid environment surrounding the fibre network, we directly probe the mechanisms governing inter-fibre adhesion. The results show that solvents capable of disrupting hydrogen bonding produce a pronounced loss of strength, whereas changes in ionic strength and electrostatic screening have only minor effects. These findings provide strong experimental evidence that hydrogen bonds constitute the dominant contribution to paper strength, in agreement with the classical model proposed by Campbell in 1933, but in contrast to more recent interpretations that downplay their significance. Hydrophobic interactions and van der Waals interactions appear to contribute secondarily to fibre bonding, although to a smaller extent than hydrogen bonding. The observed trends further reveal striking parallels between the loss of strength in different liquid environments and the hornification phenomenon occurring during drying of wet pulp fibres. Together, the results establish hydrogen bonding as the central mechanism underlying paper strength and suggest a broader conceptual link between fibre bonding and irreversible structural changes in cellulose networks.

## Introduction

Few materials have shaped human civilization as profoundly as paper. Beyond its role as a medium for art, literature and information, paper is also a vital packaging material. Today, it is increasingly explored as a sustainable alternative to petroleum-based plastics, expanding into new technological applications, such as construction^1–3^.

At its core, paper is a thin sheet of fibres formed from a water suspension known as pulp. As the water drains, the fibres come into contact and bind spontaneously. Additional chemical additives and surface treatments can further strengthen these interactions. Unlike textiles or ropes, where fibres are mechanically intertwined or twisted together, the fibres in paper are only weakly entangled. In machine-made paper, they, however, show some preferred orientation due to the manufacturing process. Thus, paper gains its strength primarily not from mechanical interlocking, but from interactions between fibre surfaces^4–8^.

Although nothing in the definition of paper restricts the fibres to plant origin, the vast majority of paper is made from plant cells or fragments thereof. Consequently, cellulose is the dominant component of pulp fibres. Other constituents of the plant cell wall, such as hemicelluloses and, in some cases, lignin, are also present in the fibre structure^9^. Since certain pulp fibres almost consist of pure cellulose, the forces that hold paper together are therefore most likely dominated by interactions between cellulose-rich surfaces. Yet the exact nature of these bonds remains debated. The classical view, dating back to Campbell^10^, holds that hydrogen bonding between hydroxyl groups is the primscallanary source of paper strength, and this view still has support^5,11–13^. Others, however, argue that such bonds contribute little—or not at all—to the strength of paper, instead emphasizing the role of other intermolecular forces or mechanical interlocking^14–17^. Robertson^18^ seems to have an opinion in the middle suggesting that additional bindings to hydrogen bonds were present. What is undisputed is that the strength of paper is not fully developed until it is dried. In industrial papermaking, drying is preceded by mechanical pressing to remove water and is subsequently completed by thermal drying^14,15,19^.

The drying process itself, however, can also have detrimental effects on the fibres – a phenomenon known as hornification^20–24^. This process – often described as irreversible – reduces the ability of cellulose to adsorb water, rendering it harder, less flexible and less chemically reactive. Paper made from hornified fibres is weaker than paper made from never-dried fibres^25^. Hornification increases with higher drying temperatures^26^, and more swollen, less crystalline cellulose is more strongly affected^27^. As with paper strength, the mechanistic basis of hornification remains debated. Some researchers emphasize the formation of multiple hydrogen bonds between cellulose surfaces^11,22,28,29^, whereas others consider their involvement minor or negligible^16,20,30,31^. Newman^32^ argued for a position where hydrogen bonds occurred, but that co-crystallization played a role, and Mo and Li^24,33^ have a similar opinion.

Hemicelluloses are suggested to play a dual role in paper properties: they act as a natural binder, significantly enhancing paper strength by promoting inter-fiber bonding^34^, while simultaneously serving as a protective ‘spacer’ within the fiber wall that mitigates hornification by physically hindering the irreversible aggregation of cellulose microfibrils during drying^11^.

In a series of previous studies, evidence have been presented that hydrogen bonds are central to hornification of chemical pulps, both as a driving force during drying—where hydrogen bonding contributes to capillary forces and brings hydrogen-bond-rich surfaces into close contact—and in determining the properties of the dried material, where multiple hydrogen bonds render the structure hard and relatively inert^28,35^. However, hydrophobic interactions, and van der Waals forces, also appear to play a role, particularly in swollen and less crystalline cellulose^27^. At higher drying temperatures, covalent bond formation, such as ether linkages, may also contribute^25,26,36^.

Paper is typically weak to water and thus exhibits lower strength under wet or humid conditions^37^, which suggests that water disrupt critical interactions between fibres and possibly also between cellulose surfaces inside the fibres. The mechanisms are suggested to in much the same ways as it enables development of sheet strength and hornification. A deeper understanding of molecular interactions is essential for developing stronger paper products and optimising manufacturing processes. Upon rewetting, water disrupts hydrogen bonds and plasticizes the fibre wall, leading to a dramatic reduction in tensile strength unless additional stabilizing mechanisms are present^38,39^. Permanent wet strength is commonly achieved through the addition of reactive wet-strength resins, such as polyamide–epichlorohydrin (PAE), which form covalent cross-links within and between fibres, thereby stabilizing the network under wet conditions^5,40,41^. In addition to chemical cross-linking, wet strength is strongly influenced by fibre morphology, degree of refining, fibre swelling capacity, sheet density, and fibre surface chemistry^42–44^. Fibre swelling and flexibility are particularly important, also here is a connection to hornification, as they govern the formation and consolidation of inter-fibre contact areas during sheet drying^45^.

Due to its connection to fibre swelling and flexibility, hornification plays a significant role in determining both dry and wet strength properties^11,25^. Since it is associated with closure of nanopores within the fibre wall, increased internal hydrogen bonding, and reduced accessibility of hydroxyl groups, which collectively limit fibre re-swelling and conformability upon rewetting^11,36,42,46^. As a consequence, hornified fibres typically exhibit decreased bonding potential and reduced stress transfer efficiency within the fibre network, thereby negatively affecting strength development^47^. Because wet strength depends not only on chemical cross-linking but also on the integrity and recoverability of the fibre network, understanding the interplay between hornification, fibre swelling behaviour, and inter-fibre bonding remains essential for optimizing paper performance in moisture-rich environments.

One possibility is that hornification and the development of paper strength are closely related phenomena. The same types of interactions that cause cellulose surfaces within a fibre to bond strongly to each other may also govern adhesion between fibres in paper. In support of such an idea, Przybysz et al.^13^ made sheets from different solvents and concluded that papers made from water were considerably stronger than papers made from more apolar solvents, suggesting a central role for hydrogen bonds. Other possible types of bonds that might be involved are interactions between apolar surfaces, i.e., hydrophobic interactions and van der Waals bonds – more disordered cellulose exposes hydrophobic surfaces^27^. Electrostatic interactions have also been suggested to be indirectly involved in paper strength, by influencing swelling and fibre flexibility^48,49^. Torgnysdotter and Wågberg^50^ used chemically modified regenerated fibres as a model system and concluded that high salt concentrations affected paper strength negatively, due to decreased swelling of fibre surfaces.

Robertson^18^ tried another approach and wetted standard blotting papers with various solvents and found that polar solvents as water and DMSO weakened the paper much more than apolar solvents such as heptane and silicon oil, with alcohols etc. in between.

In this study, the effect on paper strength of wetting paper with different solvents that influence hydrogen bonding as well as other intermolecular interactions is investigated. For this purpose, laboratory handsheets were prepared from refined softwood kraft pulp using standardized sheet-forming procedures. The sheets were dried under standard conditions and systematically investigated to find the effect of solvent properties on mechanical performance in wet conditions.

Wetting experiments were conducted using a series of solvents selected to vary in molecular size, polarity, and hydrogen-bonding capability. The different solvents enabled controlled variation in their ability to disrupt inter- and intra-fibre hydrogen bonding. After equilibration in each solvent, wet tensile testing was performed.

This experimental approach allowed correlation of wet strength retention with solvent molecular characteristics; we were able to assess the relative importance of hydrogen bonding and solvent accessibility. The results provide insight into how solvent–cellulose interactions at the molecular scale translate into macroscopic mechanical performance, and they offer a framework for understanding wet strength behaviour beyond conventional aqueous systems.

## Materials and methods

### Pulp

A fully bleached, mill-refined (21°SR) and never-dried softwood kraft pulp was used in this study. The pulp was provided by Gruvön Mill, Billerud, Grums, Sweden, and is a commercial papermaking pulp, consisting of ~ 70% Norwegian spruce (*Picea abies*) and ~ 30% Scots pine (*Pinus sylvestris*). The hemicellulose content of this quality of pulp is normally in the span of 5–12% according to the manufacturer.

### Chemicals

The chemicals that were used for the wet tensile strength tests in the study are shown in Table 1.

**Table 1:** Chemicals for wet tensile strength tests.

Chemical	Relative polarity^28^	Structure	Assay	Distributor
Water	1		–	–
Methanol	0.762		> 98.5%	VWR
Ethanol	0.654		99.9%	VWR
Acetone	0.355		> 99%	VWR
2-Propanol	0.586		> 98%	VWR
Ethyl acetate	0.228		> 99%	VWR
n-Heptane	0.012		99.8%	VWR
Magnesium sulphate-hexahydrate	n.a		min 99%	Riedel-de Haën
Sodium chloride	n.a		min 99.8%	Sigma-Aldrich
Guanidine thiocyanate	n.a		99%	Thermoscientific
Urea	n.a		100%	VWR

### Sheet preparation

All pulp sheets in the study were prepared in accordance with ISO 5269-1. The grammage of the sheets was however aimed towards 100 g/m^2^, instead of 60 g/m^2^ as stated in the standard. This was to avoid questionable test results, due to too fragile and weak test samples when soaked in different solutions. Finally, the produced sheets were then stored at 23 ± 1 °C and 50 ± 2% relative humidity (ISO 187), for at least 24 h before analysis.

### Sample preparation, wetting and tensile strength testing

**Fig. 1:**
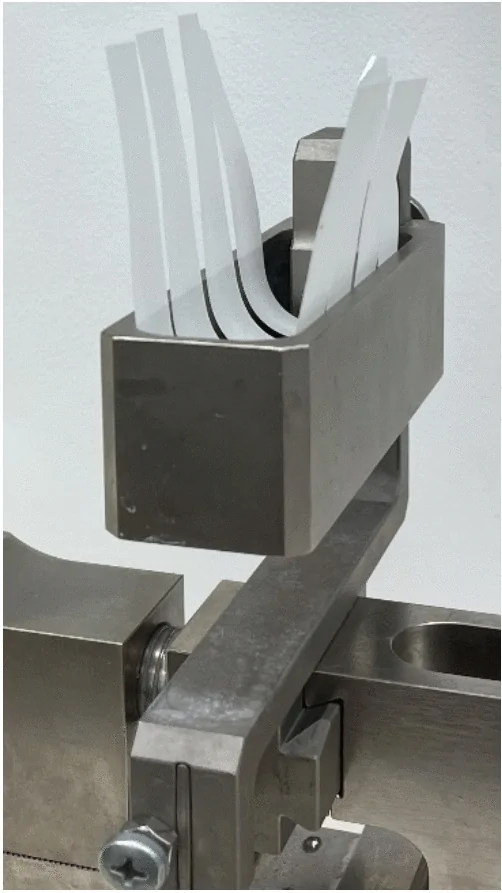
A Finch cup immersion device was used to wet part of the samples in the chemicals shown in Table 1.

Rectangular test pieces, 120 × 15 mm, were cut from the produced pulp sheets, to be used for the tensile strength analysis. Before the tensile strength testing with the wetting agents methanol, ethanol, acetone, 2-propanol, ethyl acetate and n-heptane, the sample strips were immersed and soaked in the liquid concerned. A Finch immersion device (described in ISO 12625-5) was used to wet the samples, Fig. 1, in such way that the strips only partially (ca 2–3 cm) were soaked. This to avoid wetting the clamped part of the samples. Before the samples were mounted in the tensile strength tester, they were slightly pressed between two pieces of blotting sheets to remove any excessive liquid.

All tensile strength tests using wetting agents methanol, ethanol, acetone, 2-propanol, ethyl acetate and n-heptane were performed in accordance with ISO 12625-5 using a L&W Alwetron TH1, and the tensile tests using water and wetting agents magnesium sulphate-hexahydrate, sodium chloride, guanidine thiocyanate and urea, were performed in accordance with ISO 1924-3 using a Zwick Roell Z005, with the exception that the width of the sample strips used was 15 mm instead of 50 mm. At least ten strip samples were analysed for each wetting agent used. The reason for using two tensile test devices was that the L&W Alwetron TH1 was not able to measure the relatively low tensile strength of the water- and salt treated samples.

The soaking time needed for complete wetting differed between the wetting agents. For the samples treated with the wetting agents methanol, ethanol, acetone, 2-propanol, ethyl acetate and n-heptane, it was dependent on the different solutions and was determined when the tensile strength index was not significantly decreased, i.e. the soaking agent and the fibres had reached an equilibrium. The soaking time for water and the wetting agents magnesium sulphate-hexahydrate, sodium chloride, guanidine thiocyanate and urea, however, was set to 15 s as the equilibrium of the tensile strength decrease almost immediately was reached after immersion.

### Sample preparation and performance of a liquid sorption test

**Fig. 2:**
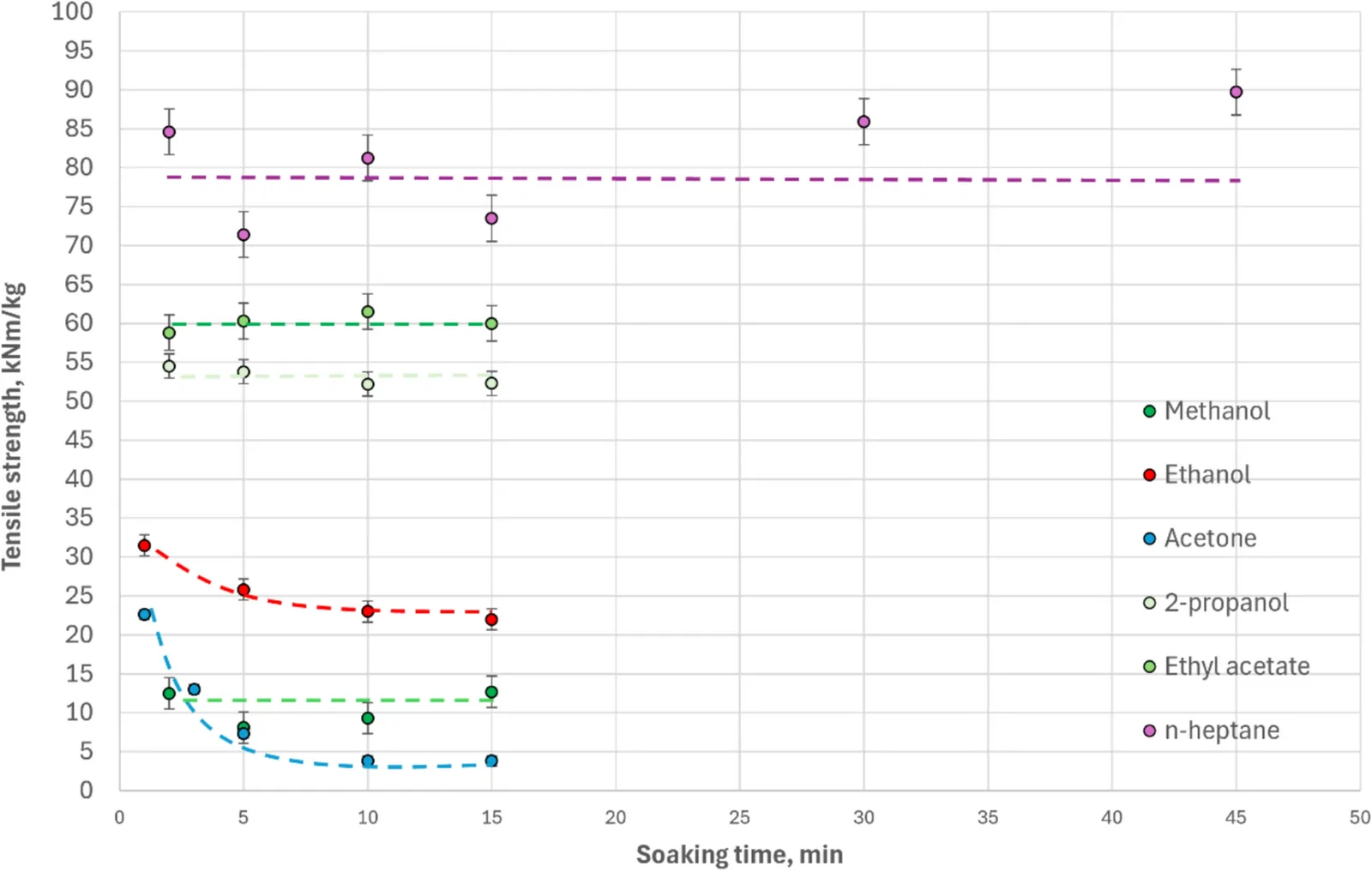
The soaking time of the samples for the different wetting agents methanol, ethanol, acetone, 2-propanol, ethyl acetate and n-heptane. The dotted lines are added for visual guidance.

Due to the different wetting times of the solvents and salt solutions, samples were cut from pulp sheets with well-defined sheet areas. The stripes were then weighed and put in a container with each of the wetting agents that differed in wetting times: water, methanol, ethanol, 2-propanol, acetone, ethyl acetate and n-heptane. Three stripes were put in each bottle and a timer was started. The wetting time for each solvent was determined in accordance with the soaking test performed prior to the tensile strength testing (Fig. 2).

When the determined time for each wetting test was reached, one of the stripes was removed from the bottle and put between two blotting sheets to remove any excess liquid and was then quickly weighed. This procedure was repeated for the other two stripes in the bottle, before the samples of the remaining wetting agents were tested in the same way as described above.

The weight of every stripe was recorded, and the liquid sorption was calculated as the weight difference between the soaked and the dry stripe divided by the stripe’s area (Eq. 1).

1 $$\frac{{Stripe}_{soaked}- {Stripe}_{dry}}{area \, of \, stripe}=g/{m}^{2}$$

## Results and discussion

Laboratory sheets, handmade papers, were prepared from fully bleached softwood kraft pulp fibres in unbeaten form. Since no strength additives were used, this system consists chemically predominantly of cellulose, with smaller amounts of hemicelluloses, mainly xylan and mannan. The lignin content is very low.

It is well known that paper loses a significant fraction of its strength when wetted with water. This observation can in itself be taken as an indication of the involvement of hydrogen bonds in paper strength, since water easily disrupts such bonding. However, a liquid may also act as a general spacer between fibres, thereby reducing mechanical integrity independently of specific hydrogen-bond disruption, the strength reduction in that case will be depending on how well the solvent can penetrate the fibre network and access the bonding sites. The sheets were wetted with a number of liquids (Table 1) of varying polarity and hydrogen-bonding ability, ranging from water (high polarity) to n-heptane (low polarity). Before wet strength analysis, the required soaking time for each wetting agent was determined – defined as the time when the liquid had fully penetrated the sample and equilibrium between the fibre web and the wetting agent was reached. Equilibrium was considered reached when no further significant decrease in tensile strength index was observed (Fig. 2); thus wetting was assumed to be completed.

**Fig. 3:**
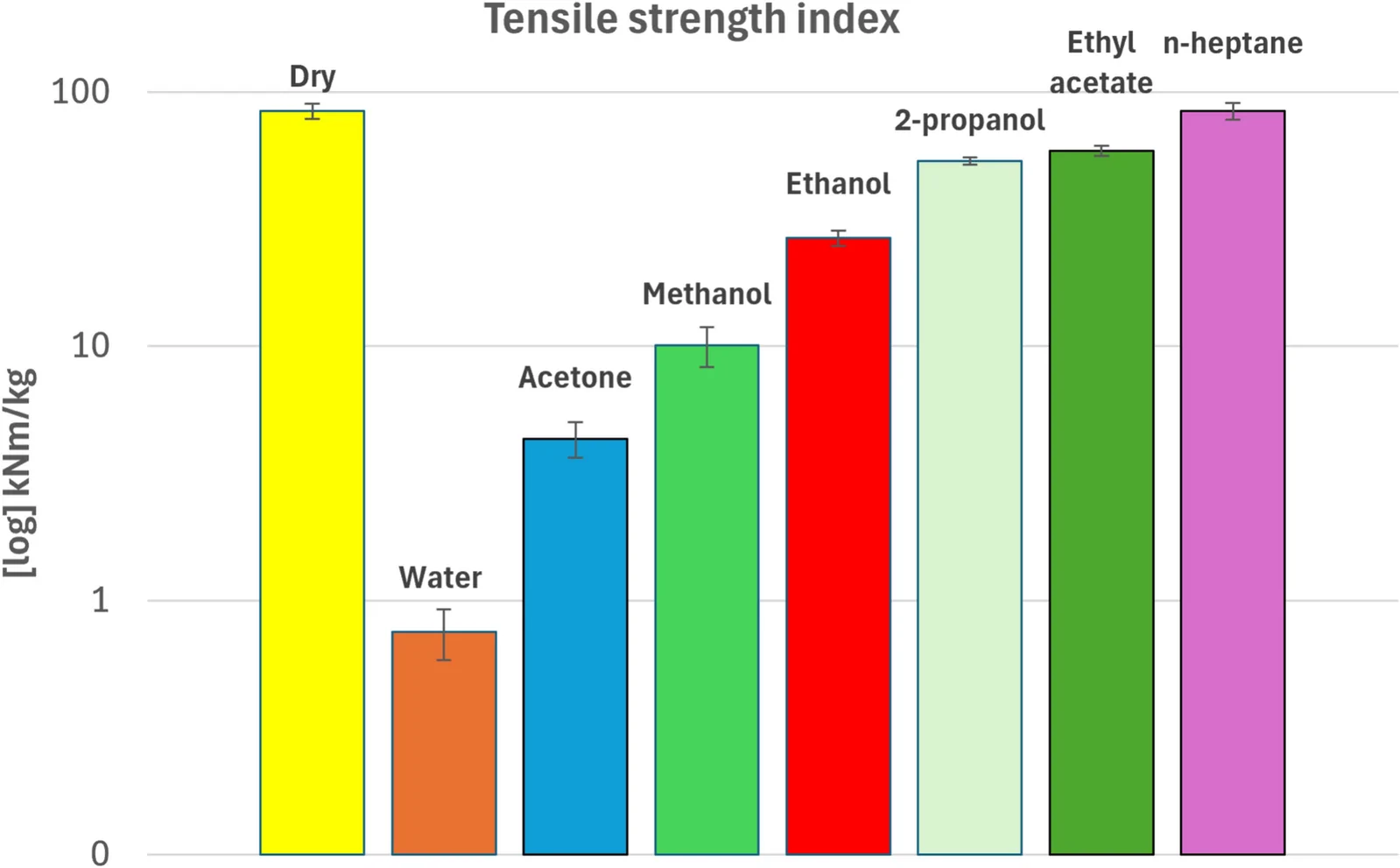
Tensile strength index for sheets of pulp wetted with different liquids, and an unwetted control.

**Fig. 4:**
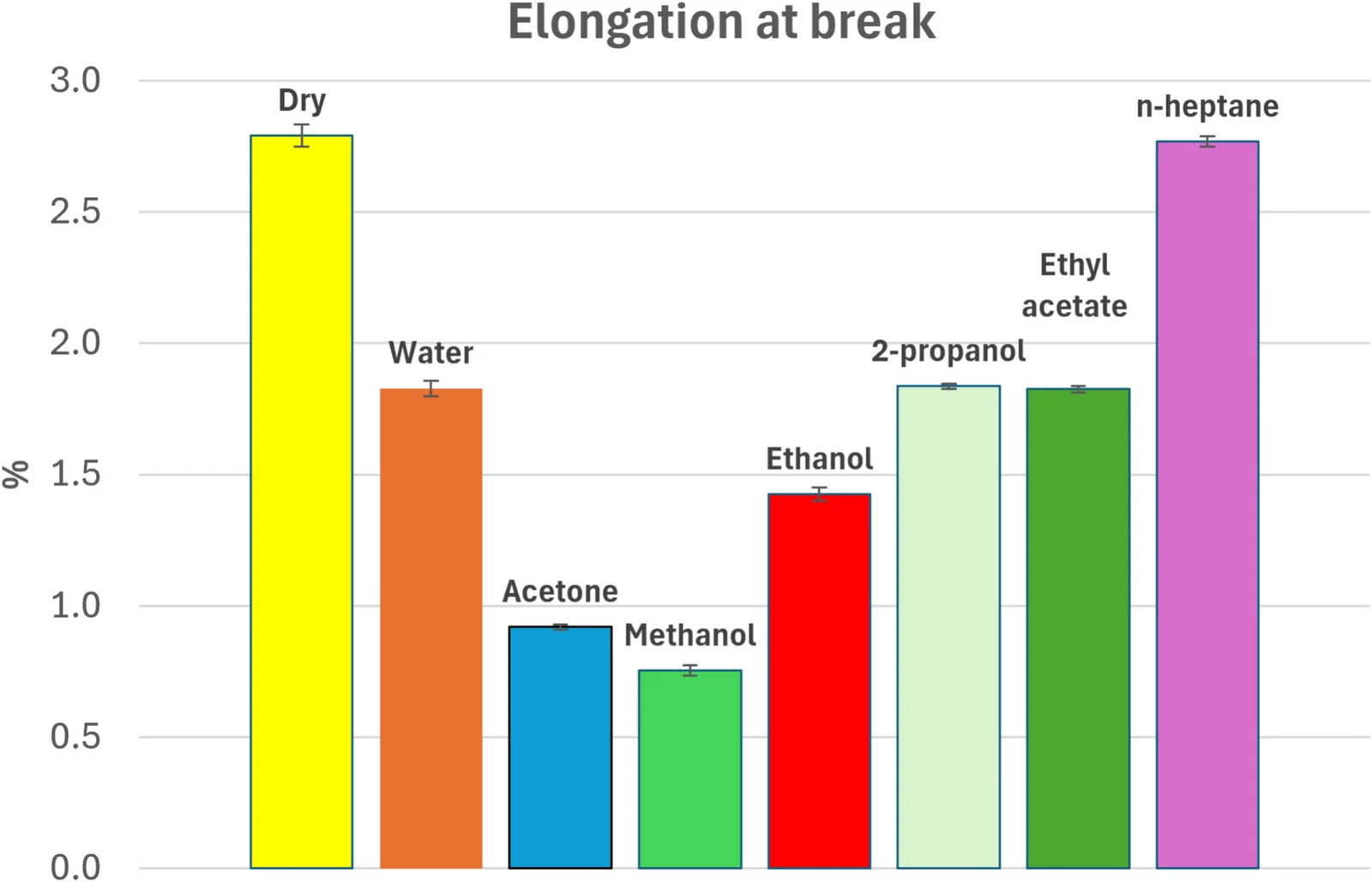
Elongation at break for sheets of pulp wetted with different liquids, and an unwetted control.

**Fig. 5:**
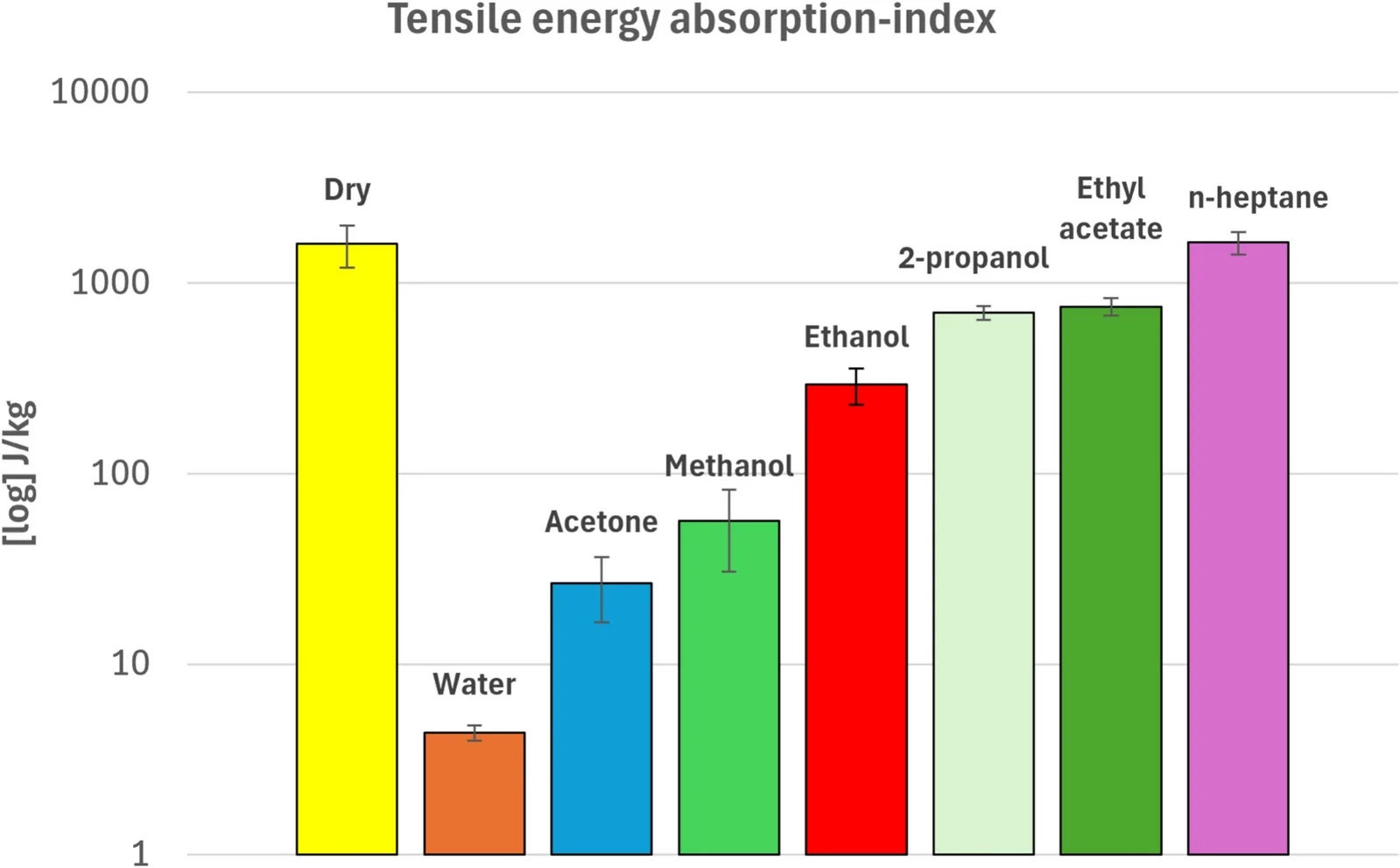
Tensile energy absorption (TEA) index for sheets of pulp wetted with different liquids, and an unwetted control.

Tensile index, elongation at break, and tensile energy absorption were measured for sheets soaked with the different solvents. The results are presented in Figs. 3, 4 and 5. Water weakened the paper by far the most for all strength parameters measured, followed by acetone and methanol. The apolar solvent n-heptane did not affect sheet strength significantly, while 2-propanol and ethyl acetate caused only limited weakening. Ethanol showed an intermediate effect.

**Fig. 6:**
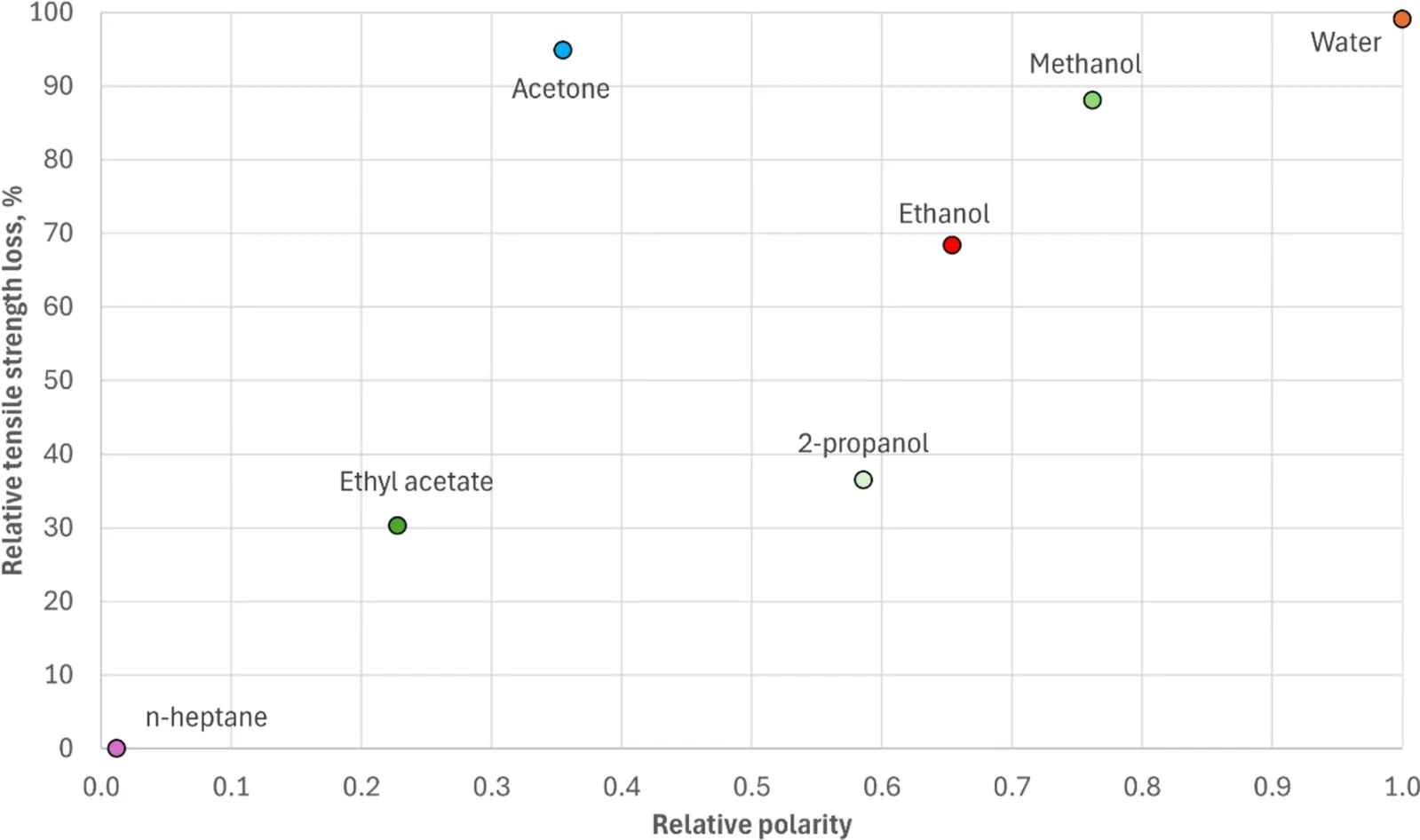
Relative polarity versus relative tensile strength loss for pulp treated with water and the wetting agents methanol, ethanol, acetone, 2-propanol, ethyl acetate and n-heptane.

Figure 6 shows a plot of tensile strength versus the relative polarity of the solvents, indicating a relationship between polarity and strength loss. However, acetone weakens the paper considerably more than expected from its polarity alone. This data have similar trends as the results by Robertson^18^, but the relative effect in this early study seems to be somewhat weaker than in our study. This might possibly be due to that Robertson used standard blotting paper, and softwood kraft pulp was used in this study, since blotting paper is quality made for adsorbing liquid, and generally are made of pulps with very high cellulose content. Differences both in hemicellulose content and in the properties of the sheets are therefore likely.

These observations from the data are largely interpreted in line with the classical view of Campbell^10^, in which hydrogen bonding plays a dominant role in paper strength. Small molecules that readily disrupt hydrogen bonds – water, methanol and acetone – weakened the paper more than n-heptane, which lacks hydrogen-bonding ability and caused almost no change to the strength of the paper sheets. Ethyl acetate, and 2-propanol which is a larger secondary alcohol, in contrast to the primary alcohols methanol and ethanol, caused only limited weakening.

Why does acetone weaken the paper more efficiently than its polarity would predict? This is indicating that more than polarity (Fig. 6) will affect the strength reduction, possibly due to the fact that acetone is very similar to the water molecule, both in size and the fact that it can interrupt hydrogen bonds. Acetone can also act as a proton acceptor in hydrogen bonds, but lacks the ability to act as a proton donor. This is in contrast to water, ethanol and methanol and even 2-propanol, which can both donate and accept protons. The latter molecules therefore have the ability to form hydrogen bond chains that can crosslink cellulose chains and contribute to some wet strength in paper. Acetone, being unable to participate in such chains, may instead disrupt inter-fibre hydrogen bonds more effectively without competing crosslinking. Such hydrogen bond chains have been suggested to be important in hornification mechanisms^35^.

**Fig. 7:**
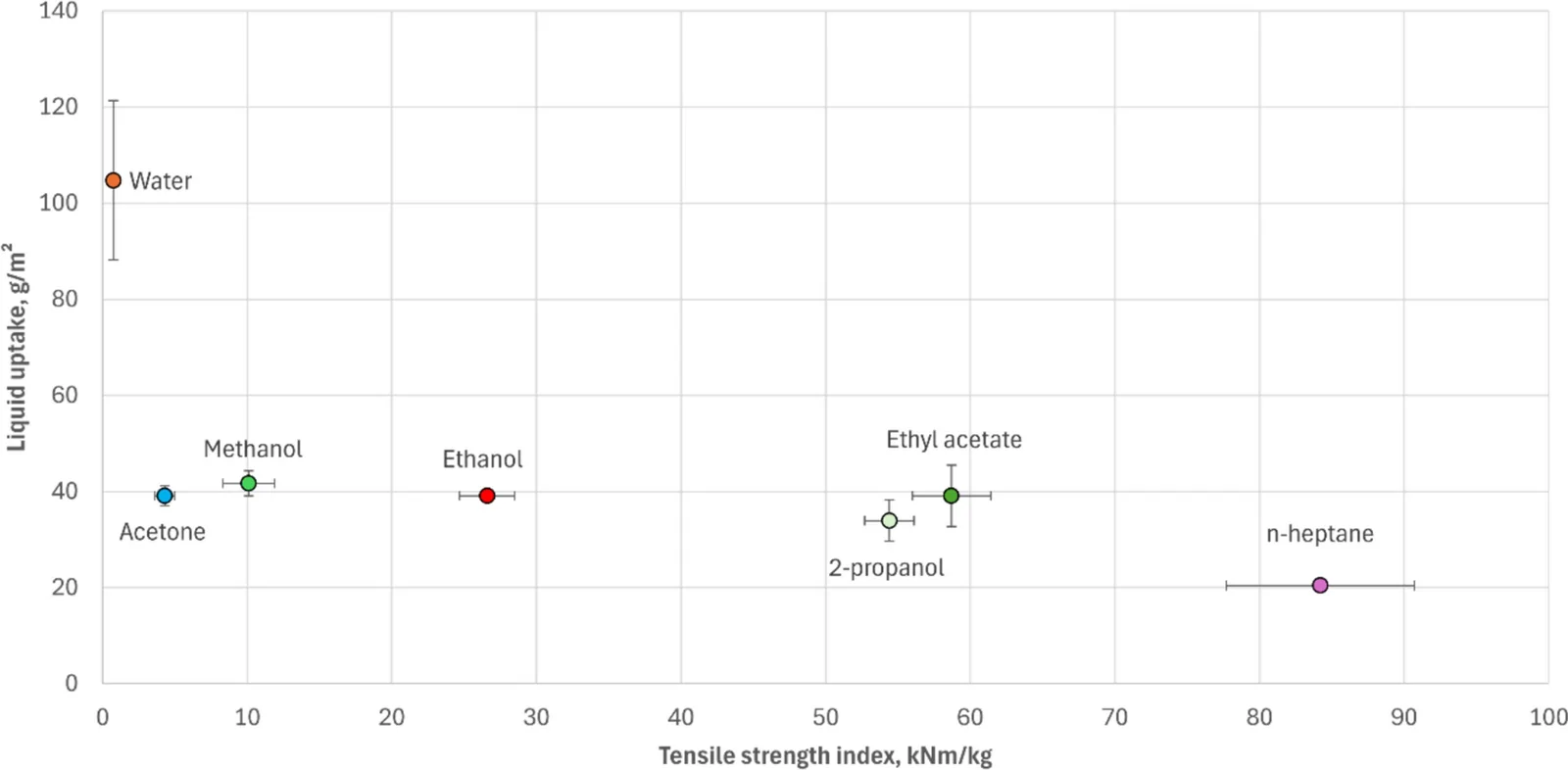
Tensile strength index versus liquid uptake for different solvents. The wetting time for each solvent is shown in Table 2.

**Fig. 8:**
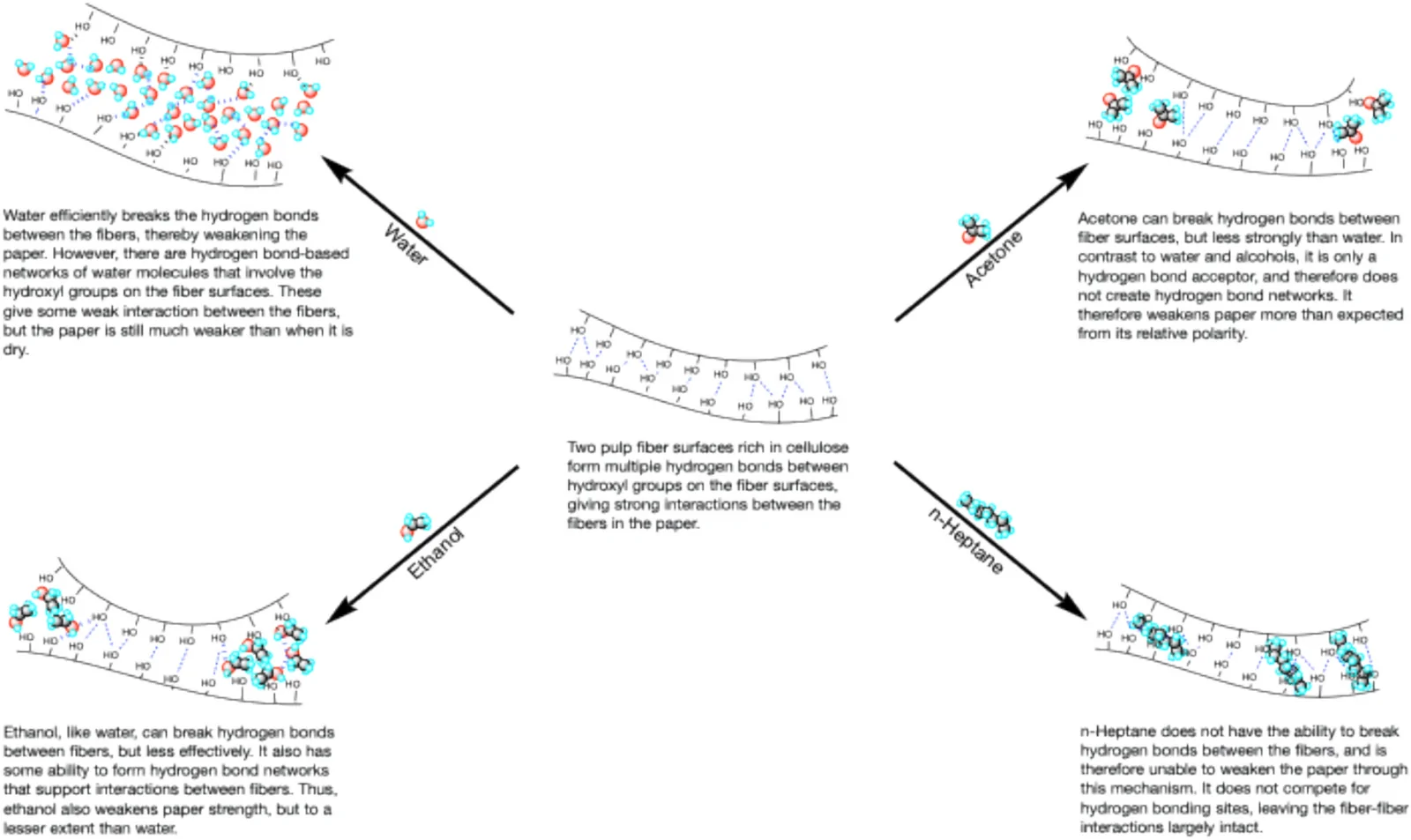
Hypothetical explanation for different responses of wetting of paper, with different solvents.

Since it is also observed that none of the solvents are effectively wetting the paper sheets, apart from water (Table 2), there is no clear indication that the wetting ability relates to the strength differences (Fig. 7) indicating that the weakening of paper is more than just a wetting phenomenon. Figure 8 presents a hypothetical explanation for how the different solvents affect hydrogen bonds in paper.

**Table 2:** The average liquid uptake values for the samples at different wetting times.

Wetting agent	Average liquid uptake values, g/m^2^	Standard deviation, g/m^2^	Wetting time, min
Water	104.7	16.6	1
Methanol	41.7	2.6	5
Ethanol	39.1	0.0	10
2-propanol	33.9	4.3	10
Acetone	39.1	2.1	10
Ethyl acetate	39.1	6.4	10
n-heptane	20.4	0.3	30

Notably, the order in which the solvents weaken the paper is similar to the order in which they cause hornification when pulps are dried from them^28^, and the strengths of sheets formed from different solvents^13^. This is consistent with the idea that paper strength is related to hornification, i.e., that hydrogen bonds and water chains play key roles^11,35,51^.

However, although hydrogen bonds appear to play a dominant role in paper strength, other bonds may also be involved. Examples include interactions between hydrophobic surfaces – cellulose naturally contains hydrophobic surfaces, and these can be increased when cellulose is swollen^27^. Under dry conditions, these interactions are dominated by van der Waals forces. In the presence of water, hydrophobic interactions – which are entropically driven due to the reorganization of water molecules around nonpolar surfaces^52^ – also contribute. Also, purely electrostatic interactions such as salt bridges and ion dipole interactions is a theoretical possibility.

**Fig. 9:**
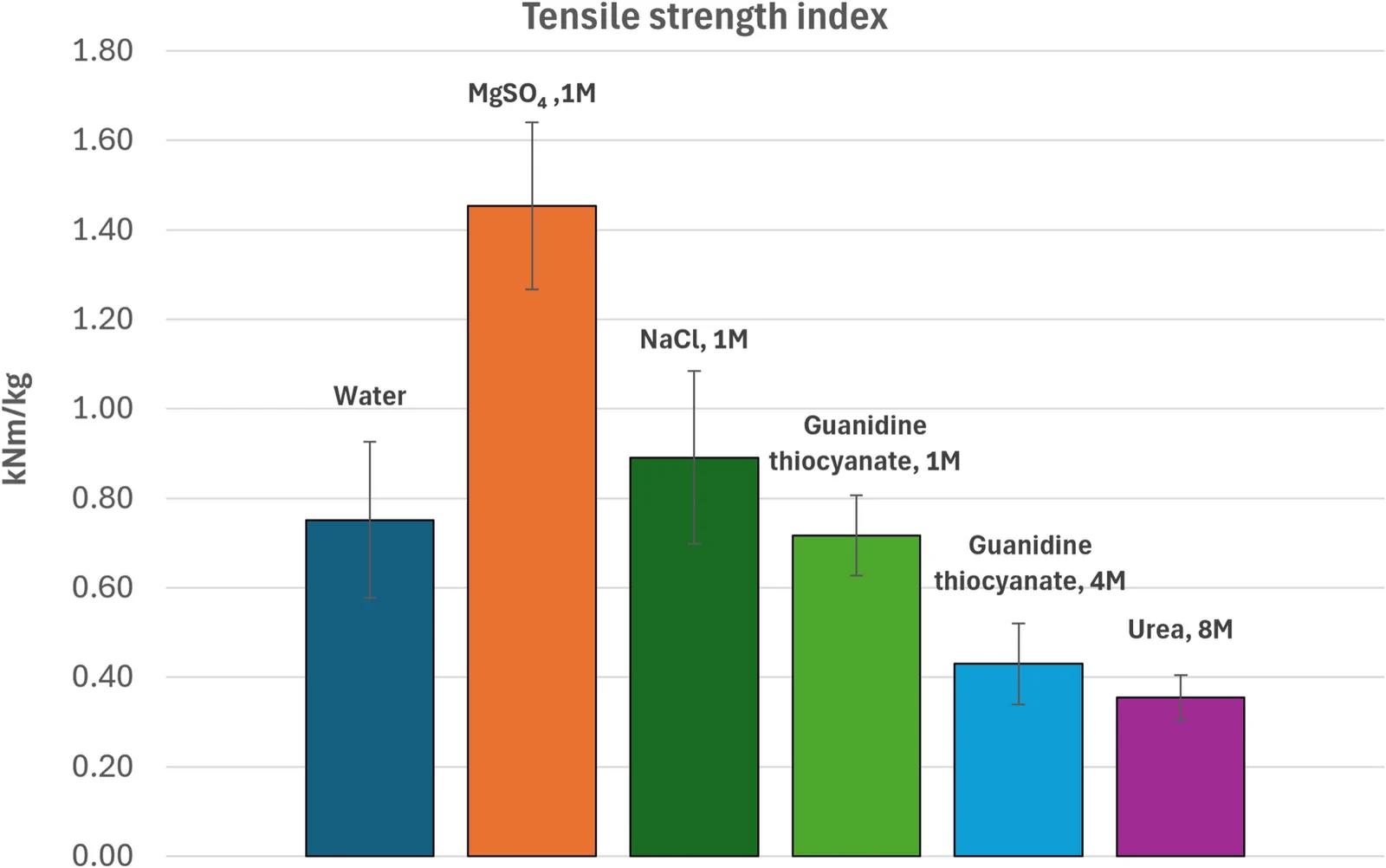
Tensile strength index for sheets of pulp wetted with different solutions of salts and urea in water and control with pure water.

**Fig. 10:**
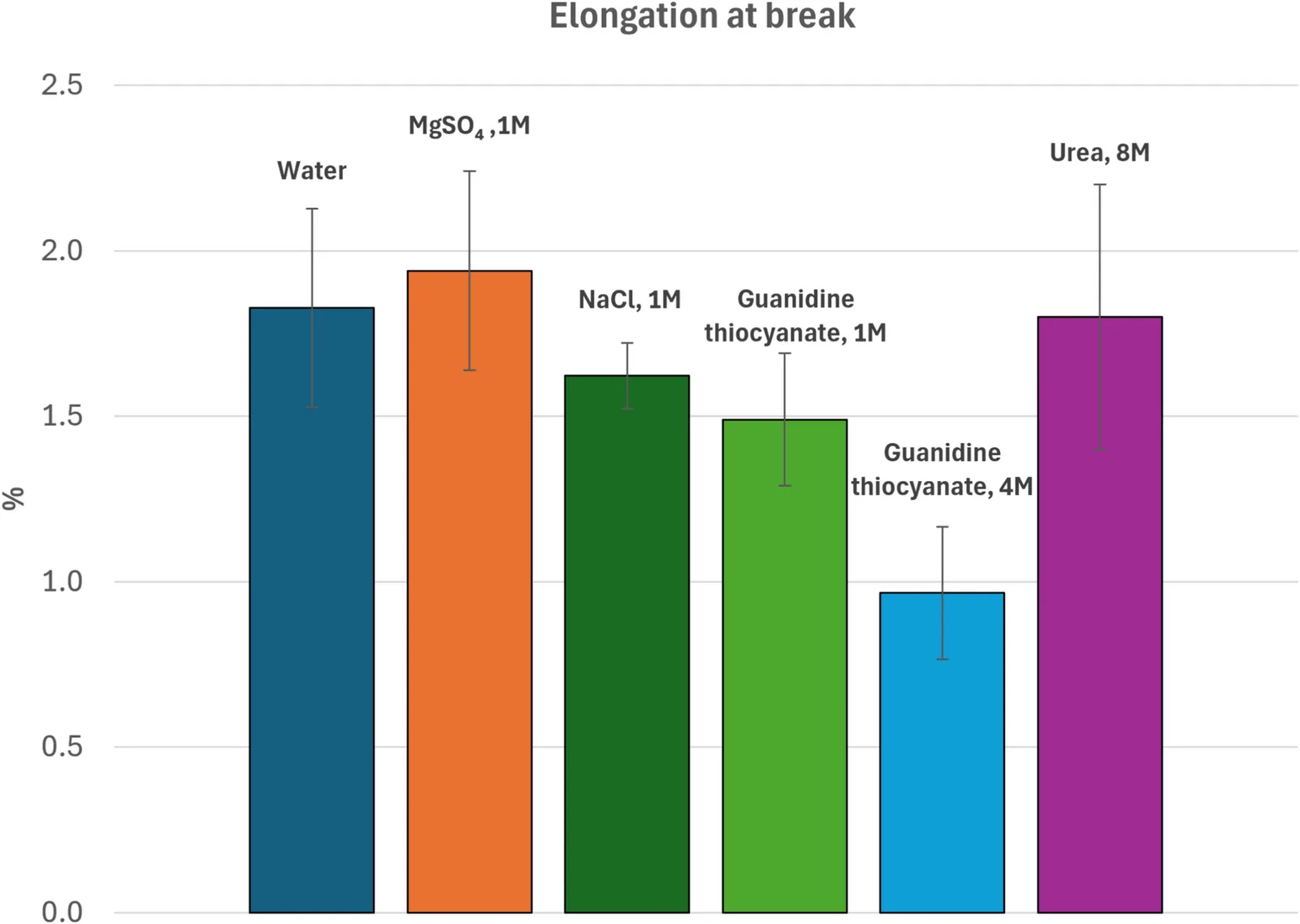
Elongation at break for index for sheets of pulp wetted with different solutions of salts and urea in water, and control with pure water.

**Fig. 11:**
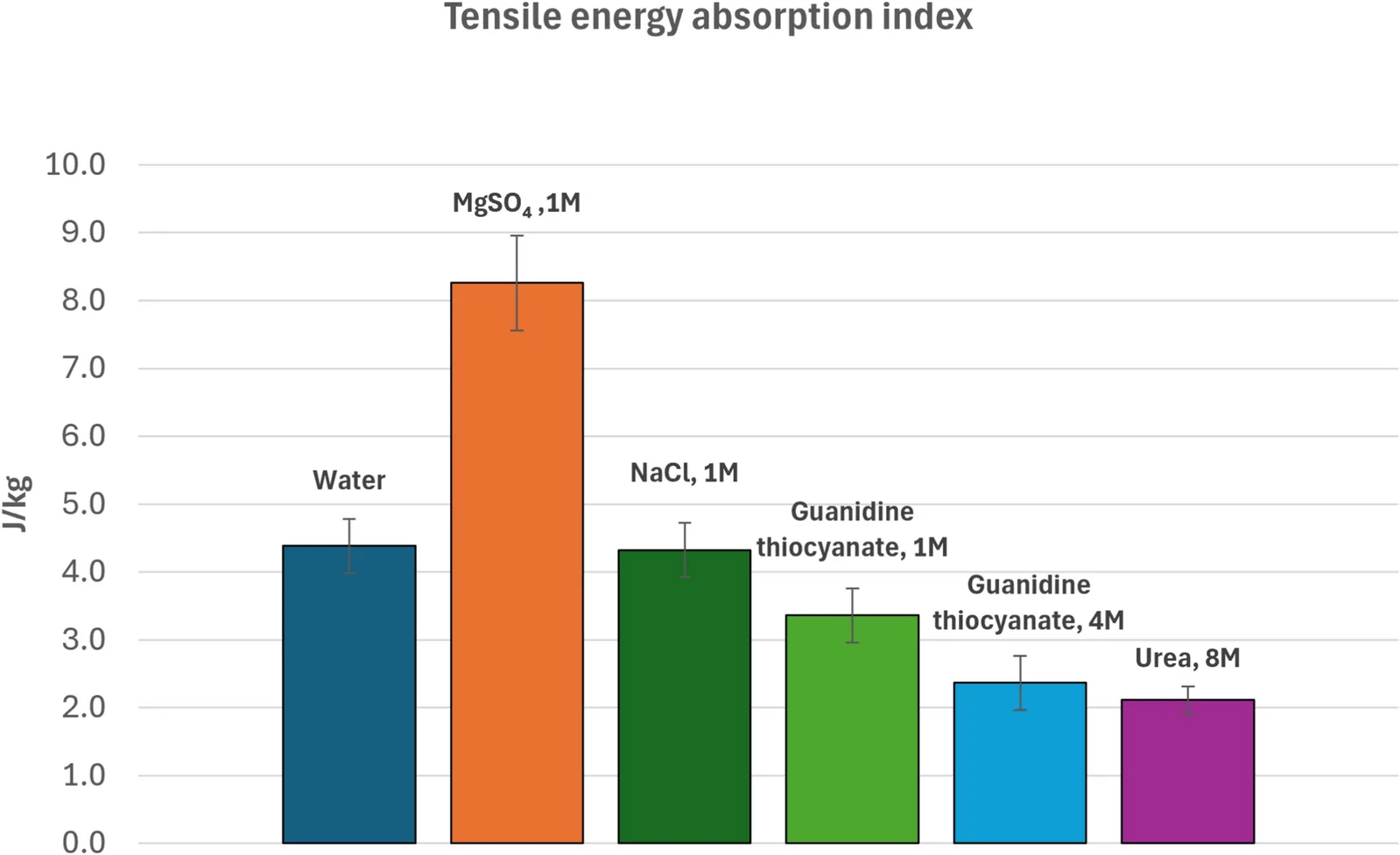
Tensile absorption energy (TEA) index for sheets of pulp wetted with different solutions of salts and urea in water, and control with pure water.

Addition of salts to water can either increase or decrease hydrophobic interactions. Salts that increase hydrophobic interactions are called cosmotropic (structure-making), whereas those that decrease them are called chaotropic (structure-breaking). Salts are organized according to this effect in the Hofmeister series^53,54^. We used magnesium sulfate (MgSO_4_), a cosmotropic salt; sodium chloride (NaCl), which has a weak effect on hydrophobic interactions but efficiently weakens electrostatic interactions; and guanidinium thiocyanate (GuSCN), a chaotropic salt. The standard concentration was 1 M. For the chaotropic salt, a higher concentration (4 M) was also tested. The strength properties were compared with sheets wetted with pure water. Results are shown in Figs. 9, 10 and 11.

The results showed that the cosmotropic salt (MgSO_4_) weakened the paper less than pure water. NaCl had no strong effect, although a slightly higher strength may be present. The chaotropic salt gave significantly weaker paper, especially at the higher concentration. These data indicate that interactions between hydrophobic surfaces also play a role in paper strength.

However, it should be emphasized that even sheets wetted with the magnesium sulfate solution were much weaker than unwetted sheets, indicating that hydrogen bonds still dominate paper strength. The fact that even high sodium chloride concentrations did not weaken the paper more than pure water suggests that purely electrostatic forces – such as salt bridges or ion–dipole interactions – do not play any significant role in the interaction in the paper. However, this result does not necessarily contradict the conclusions of Torgnysdotter and Wågberg^50^ that emphases the role of charges for paper strength *during* the sheet formation (due to surface swelling) whereas this study investigate the role of forces in the *ready-made* sheet.

As a final test, we wetted the sheets with an 8 M solution of urea in water. Urea is known as an excellent solvent for polymers such as proteins, and it has been suggested to weaken both hydrophobic interactions^55,56^ and hydrogen bonds^57,58^. However, the relative importance of these effects is still debated. For cellulose, urea has been indicated to interact directly with the hydrophobic faces of pyranose rings^59,60^. This interaction could potentially weaken hydrophobic aggregation between cellulose surfaces^61^, while urea has also been suggested to disrupt hydrogen bonds in other systems^57,58^. The sheet wetted in 8 M urea was the weakest of all sheets,its strength could only be measured with great care (Figs. 9, 10 and 11). This result appears consistent with the importance of both hydrogen bonds and hydrophobic forces in paper strength, although the relative contribution of each mechanism remains unclear.

## Conclusions

The results of this study indicate that hydrogen bonds play a dominating role in paper strength, consistent with the classical model proposed by Campbell^10^, but in contrast to more recent studies that tend to diminish the role of these bonds. The hydrogen bonds may act both between fibres and between cellulose surfaces within fibres. Interactions between hydrophobic surfaces, i.e., hydrophobic interactions and van der Waals forces, also appear to contribute, albeit to a lesser extent than hydrogen bonds. Purely electrostatic interactions, on the other hand, do not seem to play any significant role. Furthermore, paper strength appears to share mechanistic similarities with hornification, as the same solvents that cause strong hornification upon drying^28^ also weaken paper, and vice versa.

## Data Availability

All data available on request.
